# The mechanism between mortality, population growth and ageing of the population in the European lower and upper middle income countries

**DOI:** 10.1371/journal.pone.0259169

**Published:** 2021-10-29

**Authors:** Goran Miladinov

**Affiliations:** Faculty of Economics at “Ss. Cyril and Methodius”, University in Skopje, Skopje, Macedonia; Szechenyi Istvan University: Szechenyi Istvan Egyetem, HUNGARY

## Abstract

This paper analyses the effect of mortality rates (under-five and adult mortality) and population growth on the population ageing in a pooled sample of nine lower and upper middle European countries. Therefore, the main goal of this research is to investigate the ageing process of the population in the context of mortality mechanisms (under five and adult mortality) and of population growth in nine European LUMIs. The analysis is implemented in terms of Pooled least squares with cross-section fixed effects methodology. The novelty used within this research is White two-way cluster standard errors & covariance. This study is based on a database from the World Bank and UN covering the period 1995–2019. The expected results are making available quantitative analysis and insights in the context of mechanisms between the ageing process of population, mortality and population growth across these European LUMIs. Results are consistent with the notion that the increasing ageing process within these countries may be a consequence of the negative impact of population growth and from the influence of adult mortality for both sexes. The research results confirm the presence of solid ties of the mechanism between mortality, population growth and population ageing. Therefore, a clear point was provided that mortality acceleration will depend primarily on the level of population growth.

## Introduction

Over time, falling fertility and reducing mortality have resulted in an ageing population. When [1] have been examining the accelerating process of population ageing, they turned their attention to the fact that this phenomenon was due to the rising life expectancy and a decrease in fertility rates. As they further state, this experience is not new for developed countries but has also lately been expanding in less developed economies and few developing countries. This represents a new social and economic reality to which society is adjusting itself. As a dominant demographic trend of this century—population ageing, as emphasized by [2], represents also an extraordinary historical achievement. Ferdynus indicates that the demographic trend of ageing of population of a given country or within a region has been most observable in the oldest continent-Europe and in Japan [3]. Many scholars see ageing of the population as the most pressing problem of current population development, especially in economically more developed countries; see [1, 2, 4]. It is because the impact of ageing on the socio-economic development and welfare system of countries has been continually expanding (e.g., public pensions and healthcare, labor force participation, economic growth, social protection expenditures, housing).

Bengtsson and Scott [5] assessed that population ageing has evolved into a worldwide phenomenon, having an effect on many newly developed and developing countries. In addition, they point out that the reason why the proportion of elderly has increased could be very obvious: the improvements in life expectancy and increasing longevity. Looking at the time to come, population ageing is expected to expand most in low and middle income countries and more slowly in countries of Western Europe where the ageing process has long since begun [6]. (It seems that the low and middle income countries are not yet prepared to meet the financial needs by the ageing process of large cohorts such as the baby boomers as well as the population of older people in employment. Thus, as stated by [6], population ageing may result in a growing proportion of older individuals living with weak welfare support and this in turn will successively tense up individuals and families who need to provide support for older people. These concerns have caused increased research and policy attention on the ageing process. The research focus of the study is to investigate the ageing process of the population in the context of mortality mechanisms (under five and adult mortality) and of population growth in nine European lower and upper middle income countries. According to the [7], lower middle-income economies are those with GNI per capita between $996-$3.896, and upper middle-income economies are those with GNI per capita between $3.896- $12.055. Therefore, the main goal of this paper is to point out the population ageing development and the effecting mechanism of the main demographic processes. According to World Bank list of economies (June, 2018) there are nine European countries in the group of lower and upper income countries: Moldova, Albania, Bosnia and Herzegovina, Bulgaria, Macedonia, Montenegro, Romania, Serbia and Turkey [7]). The World Bank uses the term country alternately with economy and it does not imply political independence but it refers to any territory for which officials report separate social or economic statistics. The states of the former Soviet Union are not included within this research study. This research refers to the political entity known as Republic of Macedonia, which declared independence in 1991, and therefore was one of the successor states after the disintegration of the Yugoslav federation. Turkey was also included within our research as European middle income country. Turkey was recognized as a candidate for full EU membership in 1999. Turkey formally opened the next stage of the accession negotiations process with the European Union on 3 October, 2005 and since then its status has not changed. In Brussels, daily contacts and representation are ensured by Turkey’s Permanent Mission to the EU headed by its Ambassador [8]. Only Moldova Republic is a lower middle income country. The other eight countries belong to the group of upper middle income countries. In addition, all of them are geographically located in East and South Europe. In terms of demographic development, the populations in these nine countries in general have a similar stage of demographic development [9–12], with a few lesser exceptions, for instance, in Turkey and Albania. Furthermore, except for income, the general specificities and characteristics of these nine evaluated countries associate their demographics especially in regard of their high levels of infant and under five mortality rates in the past period of time and still have now compared with the EU-27 average level [7, 10, 13].

The mortality transition started much earlier in some parts of the world than in others and the pace of the demographic transitions vary significantly [14]. Having in mind the whole picture of the current demographic circumstances in Europe [12], confirm the existence of demographic variations between European countries. In the past two hundred years, According to [15] Europe went through the demographic transition from high levels of fertility and mortality to low and to currently prevalent levels of birth and death rates. All these demographic processes led to lower rates of population growth and inevitably the ageing process of the population. The increased longevity, very low infant and child mortality (mortality under five), and extremely improved education and health, all of this was a part of the modernization process in Europe [15]. A population that starts a mortality transition late, i.e. when other populations or other countries are already in an advanced stage, has a higher response rate, i.e. the decline of mortality is happening more rapidly than in the other countries that were at an advanced stage. Nowadays, mortality is low in the developed world, but remains high in poor countries. The richest countries are located in the north-western part of Europe and the poorest countries are situated in the Balkans (except for Greece) and Eastern Europe [10]. It is well known that the demographic transition began around 1800 with the decline of mortality in Europe. The beginning of the demographic transition in the world for the first time occurred in the northwest part of Europe, with a secular decline of mortality around 1800 [9]. The countries of Eastern Europe and former regions of the Soviet Union are exceptions to the generally favorable trends in mortality in Europe [9]. According to [11] typical for East European countries from the period of 1921 to 2005 at the latest, including all formerly socialist countries (former Yugoslavia, Bulgaria, Romania and Moldova) as well as Turkey was the very high birth rate and a high mortality rate, mostly infant mortality. Besides the similar transitional position of these countries regarding their economic conditions, the reason why these countries were chosen within this research study is that these countries previously experienced a strong decrease in both infant mortality and under five mortality rates. However, differences between these countries may still exist, for instance: patterns of the likely future of the population ageing trends observed in today’s low and upper middle-income countries in Europe and the role of mortality rates and population growth in light of the steep ageing progress in middle income countries. This research is offering identification of the contribution of mortality on the age structures changes. Thus, the specific contribution could be seen in making available quantitative analysis and insights in the context of mechanisms between the ageing process of population, mortality and population growth across these European LUMIs (lower and upper middle income countries). The paper is organized as follows. Sections 1 and 2 introduce the research and present theoretical background and major hypotheses of this research study. Section 3 presents both the characteristics and measures of mortality rates (under five and adult mortality) and population growth, respectively. Section 4 provides data sources and methodological approaches. Within section 5 are presented the application of a panel GMM model and the main findings. Discussion part is given in the next section 6. The concluding remarks of the research are presented in section 7.

## Theoretical background and major hypotheses

In the course of the demographic transition, first mortality declined and then fertility, causing first accelerating population growth rates, and then slowing again, moving toward low fertility, longer life and an older population [9]. A long period, the trend in the death rate was determined by both the declining infant and child mortality, i.e. under-five mortality. The recent increase in the death rate and the future increase will be related with an ageing population [14]. The phenomenon of ageing population involves several circumstances. As a matter of fact [3], argues that it implies: Prolonging human life, reducing population growth rates, increasing prosperity and improving the quality of life. A key aspect of demographic research consists in studying age-specific patterns of the various demographic events since it is known that their intensity varies across the age [16].

As [17] explains, the general shape of human mortality curves may have five dominant phases in those countries for which robust data are available. Accordingly, in some instances, some of these phases may overlap or may be similar over one another and as a result, not all of the phases can be noticed in all mortality curves. For the research purposes of this paper, the first two phases are most relevant. The first phase is called “birth and childhood”. In this phase, the mortality rate descends rapidly in the first year and gradually slower thereafter, falling to a low point that historically was seen around the time of puberty, but which now appears to emerge as early as seven or eight years of age. As contemporary causes of death typical for this first phase [17] emphasized the vulnerability of infants and specific deficits of those with congenital conditions. When historically observed, there have been higher deaths during this period (first phase) as a result of exposure to major infectious diseases. The second phase is called:”Sexual maturity and early adult life” [17]. From puberty onwards, mortality begins to rise and with a demonstrated upward convexity of the mortality curve. It is easily seen in both sexes, particularly for men. The upward convexity appears to be a characteristic that reflects a combination of increased opportunity to experience outward dangers and worsened risk-taking behaviors. Milne [17] also mentions the other three phases: The ‘Makeham’ phase (third phase), the ‘Gompertz’ phase (fourth phase) and late life deceleration and extreme old age (fifth phase).

Evolutionary biologists, gerontologists and demographers have conceptualized the parameters of the Gompertz model incorporating two different mortality components: *mg* or the IMR, set by general sort of characteristics and local extrinsic threats that affect all ages, and the other, γ, the slope of the increase with age, set by intrinsic vulnerabilities that accumulate across adulthood due to physiological ageing [18]. Extrinsic mortality is associated with risks such as accidents, disease, climatic hardships and food shortages. These risks set the mortality “level” for all members of a population, moving the whole survival probability up or down. Intrinsic mortality is associated with the progressive physiological deterioration that begins after maturity and results in demographic ageing and with the “increase in mortality risk with age” across most of adulthood. In the Gompertz model the exponent, γ (γ*k* in GM) indicates the rate of increase in mortality with increasing adult age *x*. Hawkes et al. [15], discuss that the relationship between mortality level and rate of increase in mortality by age among populations could be strongly negative: ‘‘High mortality rates in disadvantaged populations are compensated for by low obvious ‘‘ageing rates”. The rates of increase in mortality across adulthood vary systematically so that the inverse relationship between mortality level and rate of demographic ageing in populations is expected to be unusually strong [18]. These authors make the explanation that there is general agreement by what has driven rising mortality with the rising of age. Also [19], had noted that there is considerable disagreement about the mechanisms that drive ageing. Their interest was how ageing should be measured; how extrinsic mortality shapes ageing and how to link individuals’ selection with demographic aging parameters that are often used for study of age-specific mortality. Burger and Missov [19] have defined demographic ageing as a change in the risk of death with age that usually is measured by a mortality model suitable for population data consisting of a lot of individuals or subgroups. These scholars consider that the Gompertz provides a well-grounded demographic measurement of ageing.

As known, the rate of demographic ageing refers to the slope of the mortality curve (Gompertz slope) and it represents the extent of acceleration in the mortality rate through the age. According to [20], the Gompertz’s classical law of mortality from 1825 simulates the increase in mortality rates over adulthood in an exponential pattern: *R*_*t*_ = *R*_0_*e*^*αt*^, where *R*_*t*_ is the mortality rate at age *t*, *R*_0_ is the initial mortality rate, and α refers to the rate of increase in the mortality rate, alternately described as “mortality acceleration” or the rate of demographic ageing. Zheng [20] further explains that the mortality acceleration parameter α is affected by both the variance of the frailty distribution in the population and by the initial mortality rate. Consequently, to understand the change in demographic ageing (mortality acceleration) across cohorts, there should be investigated whether different cohorts experience different mortality selection processes. The cohort evolution theories emphasize different mechanisms linking early and later life mortality, suggesting a positive correlation between these two age-specific rates of mortality. Hence, obviously, mortality selection mechanism indicates a negative correlation between the mortality level at young-age and rate of mortality acceleration. In addition, it has been pointed out that the substantial mortality declines have little effect on the average rate of acceleration of mortality during ageing [20]. In other words, despite the trend in overall mortality across cohorts, the mortality slope (Gompertz slope) appeared to be relatively stable. Anyway, as stated by Zheng [20], both theories, general theory of mortality and ageing and the theory of population heterogeneity, indicate that mortality selection operates throughout the life course and leads to an inverse relationship between young age mortality rates and the extent of mortality acceleration (until very late age) across cohorts.

From a demographic point of view population ageing is considered as one of the most typical features of the second demographic transition, as a result of which the proportion of population of older age groups is increasing. According to [21], changes in age structure measures in high-income and middle high-income countries are basically driven by changes in fertility and mortality rates. Furthermore, as claimed by [21], in the long run, if fertility and migration rates remain the same, i.e. unchanged, it may lead to a decrease in mortality rates at older age, thereby increases both the conventional proportion of the population considered as old and the population’s median age. But, on the other side, it decreases the proportion of the population who are old, measured by prospective concept and the population’s prospective median age. The prospective continuation of increases in longevity and population ageing, and the related challenges with the budget, particularly in relation to long-term care, health care, and pensions, have become serious concerns for governments in more developed countries [22]. Káčerová et al. [4]. have introduced an interesting perspective of ageing, presenting two dimensions: structural and numerical ageing. The structural ageing is mainly the result of the decrease in fertility. A decrease in the mortality of children and an increase in life expectancy result in a higher number and higher share of elderly population. Accordingly, the numerical ageing is in principle caused by a drop in mortality.

Káčerová et al. [4], further present the mechanisms between mortality, population growth and ageing process of the population: If child mortality is decreasing, most children survive, causing acceleration of population growth. During the several decades, these children reach reproductive age and with that causing further acceleration of population growth. Both events cause an initial rejuvenation of the age structure. Thus, the adult populations, i.e., those who have survived childhood are more likely to live until old and very old age. High fertility during the baby-boom years was also a contributing factor to numerical ageing, but it was not the dominant cause. As [4] explain further, the population will not increase significantly in the number of elderly people, if mortality is high and even if fertility is very high. In addition, the death rate may lead to both population rejuvenation and ageing and for that reason this is another important factor. This mechanism [4] explain very simply: If mortality in young age groups is decreasing (infant and child mortality), the population gets primarily younger and if mortality in higher age groups is decreasing, the population gets older. After displaying the overall theoretical background in this section, the major research questions related with the main research hypothesis for this research study would be the following: What demographic events have driven the changes in the rate of demographic ageing? Does a mechanism indicate that the rate of demographic ageing should be negatively affected by young-age mortality? And, does mortality acceleration in older ages is weak when young-age mortality is high and strong when young-age mortality is low? Thus, the major hypotheses for this research would be as follows: ‘‘There is association between mortality, population growth and population ageing in the European LUMIs”. Furthermore: ‘‘Mortality acceleration in older ages within these countries is affected by the main demographic events”.

## Measures of population ageing, mortality rates and of the population growth

**Fig 1:**
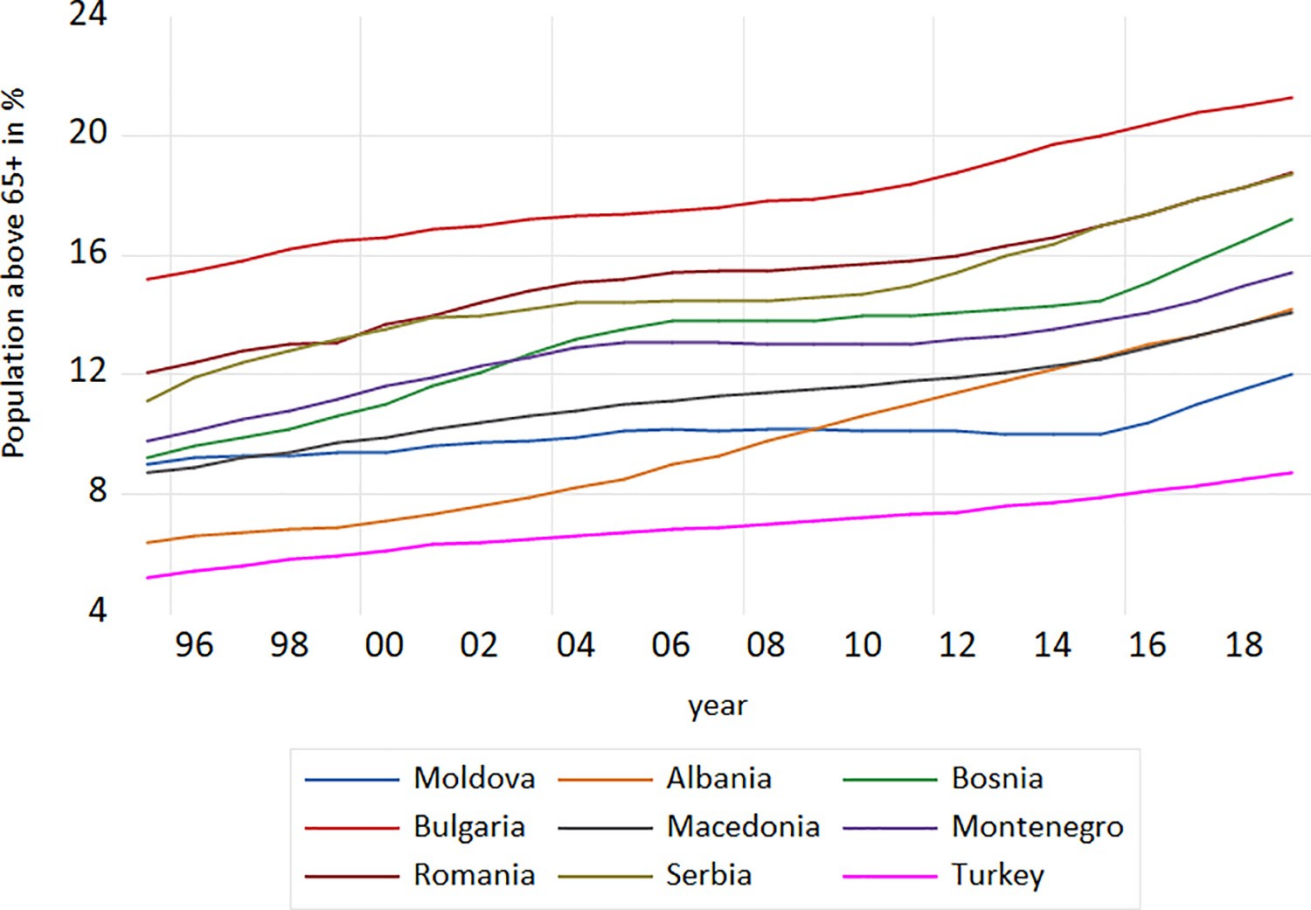
Population 65+ in the European middle income countries, 1995–2019. **Source:** Author’s design based on World Bank and UN data.

Population ageing is often measured using changes in the proportion of the population categorized as old and changes in the median age. Sanderson et al. [21], discuss about the conventional and prospective proportion of the population categorized as old. Accordingly, the *conventional proportion of the population categorized as old* is measured as the proportion of population who are above a fixed chronological age. The *prospective proportion of the population categorized as old* is the proportion of population with less than a fixed remaining life expectancy. Sanderson et al. [21], proposed a fixed remaining life expectancy of 15 years. Most measures for population ageing are based on a chronological concept of age. Researchers, governments and policy makers preferably focus on indicators, such as the proportion of the population above the age of 60 or 70, with the explanation that these chronological measures indicate the size of the dependent population [21]. It is observed that in both cases population ageing has been seen as a reduction in the proportion of the population that is under 20, and in an increase in the proportion over 64 [23]. In the less developed countries, the process of population ageing has already started, and over the next decades the largest change would be expected to be the reduction of the proportion of the young population. With the increase of longevity, the average age of the population increases by increasing the age at which each person is old relative to the persons that are young. Within our research, the age of 65+ as a conventional old-age threshold was used. The percentages of *population above 65+* in the nine European middle income countries within our research study are provided in Fig 1.

A large body of evidence document that macroeconomic conditions are crucial determinants of individual health, including child and adult mortality, e.g. [24–27]. However, child and adult mortality appear to be affected by the macro environment in different ways [25]. The evidence also suggests that human capital is important for adult longevity since adult longevity depends on the ability to cure diseases and it is related to the level of medical knowledge. Cervellati and Sunde [25] have stressed out that better living conditions in terms of higher incomes, but also in terms of access to water and electricity were relatively more important for increasing the survival probability of children.

**Fig 2:**
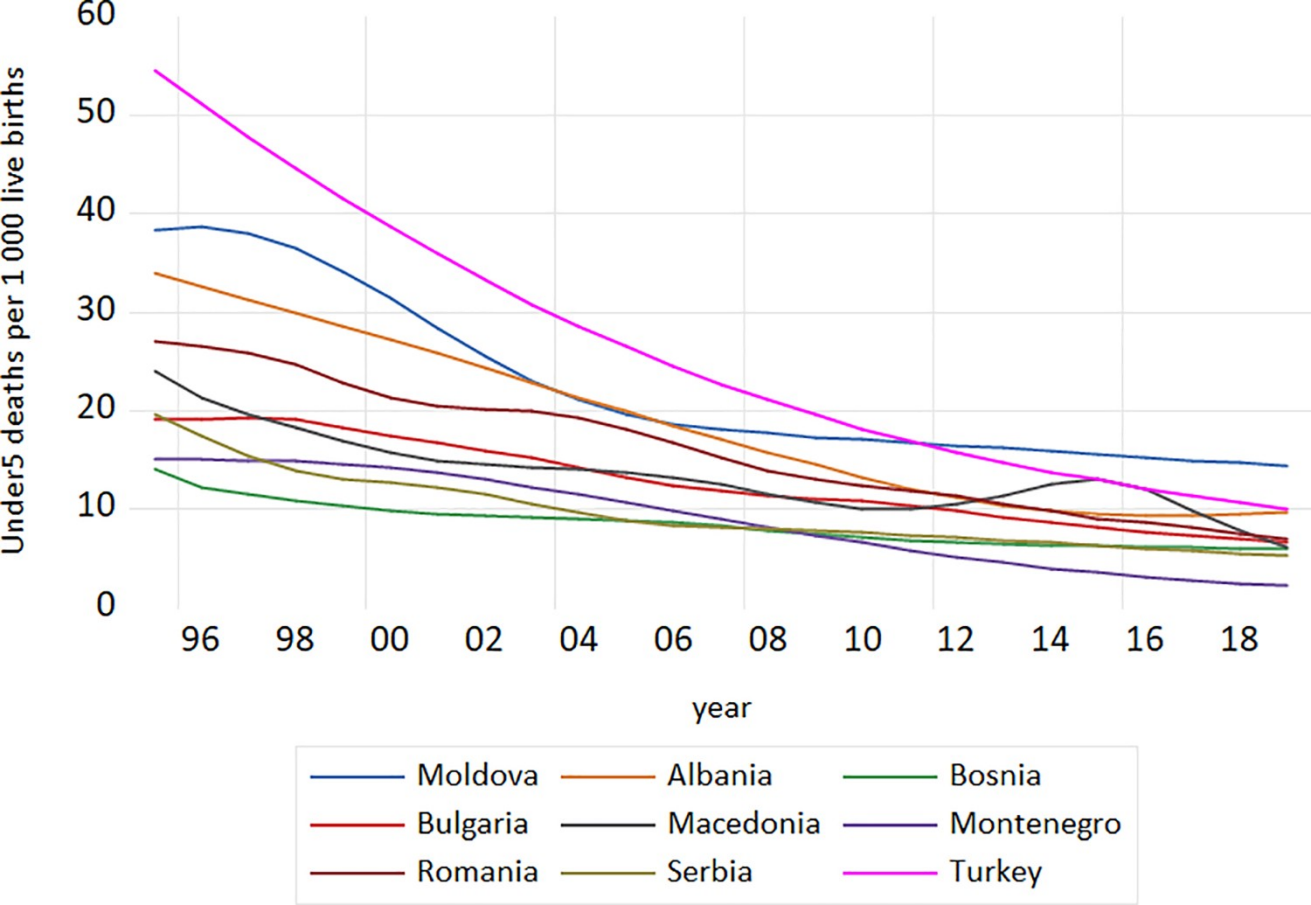
Under five mortality rate per 1000 live births in the European middle income countries: 1995–2019. **Source:** Author’s design based on World Bank and UN data.

Under-five mortality is measured as the probability of dying between birth and age 5. Under-five mortality is still considered as one of the most effective indicators of social development and health inequalities [28]. Knowing its persistently high levels in the 1990s and 2000s, and its impact on life expectancy, under-five mortality has received considerable attention and needed focus in action programs, like MDG 4 and SDG and also numerous publications and major reviews were devoted to this question [28, 29]. SDG (The Sustainable Development Goals) have purpose to put an end to the preventable deaths of newborns and children under five years of age until 2030 [29]. Particularly, the reduction of under-five mortality by two-thirds between 1990 and 2015 was the central target of Millennium Development Goal 4 of the United Nations Millennium Declaration. The Millennium Development Goal 4 target of a two-thirds reduction in under-five mortality between 1990 and 2015 has not been reached at the global scale. More recently, in the 2030 agenda for sustainable development the second target of Goal 3 was adopted in order to reduce under-five mortality to at least as low as 25 per 1 000 live births by 2030 [30]. Fig 2 presents *under-five mortality rate* in our nine middle income European countries.

**Fig 3:**
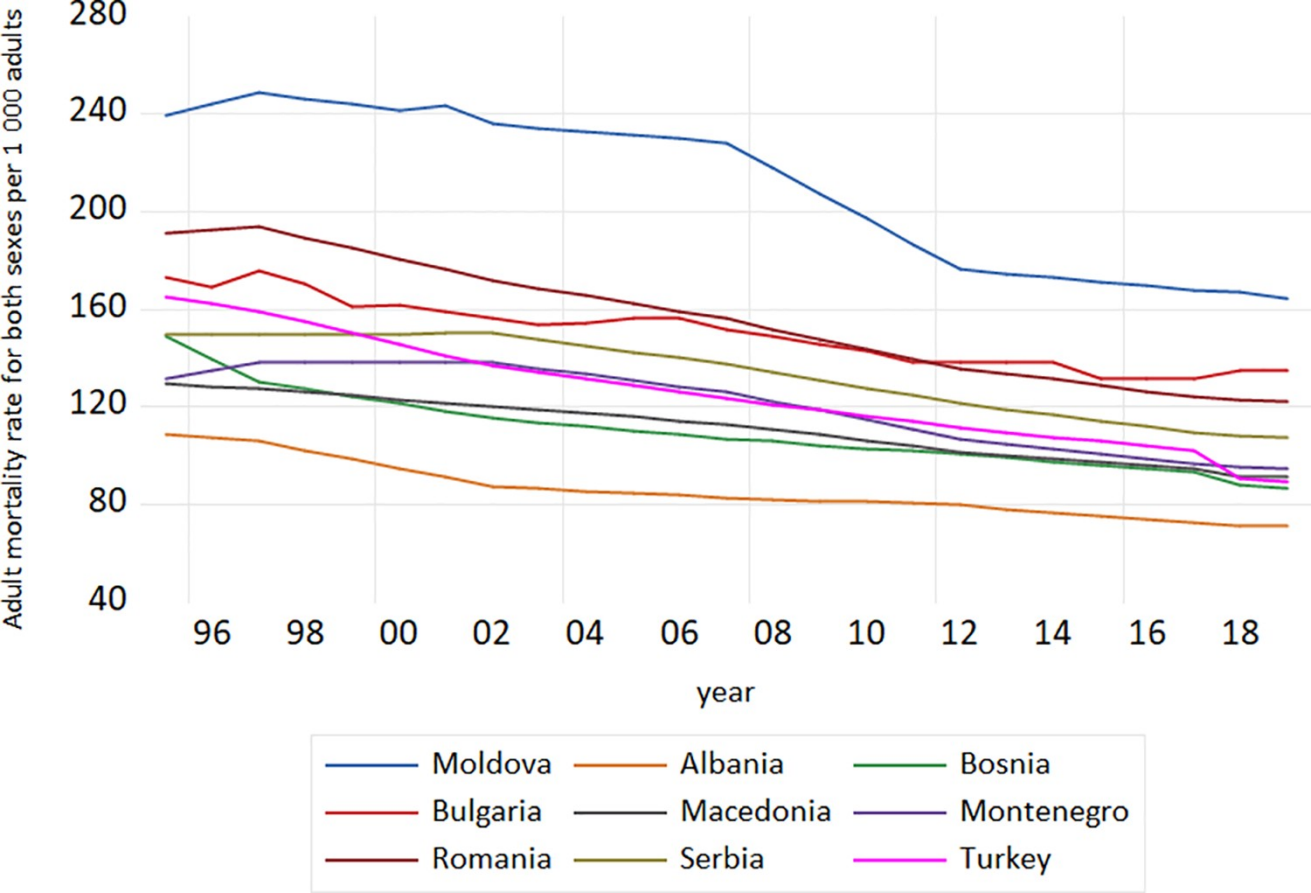
Adult mortality rate for both sexes in the European middle income countries, 1995–2019. Source: Author’s design based on World Bank and UN data.

The interest for adult mortality in low and middle income countries has long been abandoned by demographers, to some extent because data are scarce [28] but the interest has recently increased as child mortality has started to fall. To assess adult mortality, the probability of dying between exact ages 15 and 60 years was used, marked as *45q15*. According to the [30], this measure is relevant for health policy because it covers the risks of mortality, affecting young and middle-aged adults, most of whose deaths are considered to be “preventable”, such as through changes in risk behaviors (e.g., tobacco use) or by early medical intervention. Among the more developed regions, *45q15* in 2010–2015 stood at 102 per 1 000 adults in Northern America and 126 per 1 000 adults in Europe [30]. In particular, in spite of the higher level of development, the level of *45q15* in Europe was just slightly below the levels in Asia and Latin America and the Caribbean (136 per 1.000 and 138 per 1 000 adults, respectively) [30]. It is assessed that the European average was falling downwards as a result of the relatively high adult mortality in Eastern Europe, mostly among men [30]. *Adult mortality rate* for both sexes for the nine European middle-income countries, as part of this research study, is given in Fig 3.

**Fig 4:**
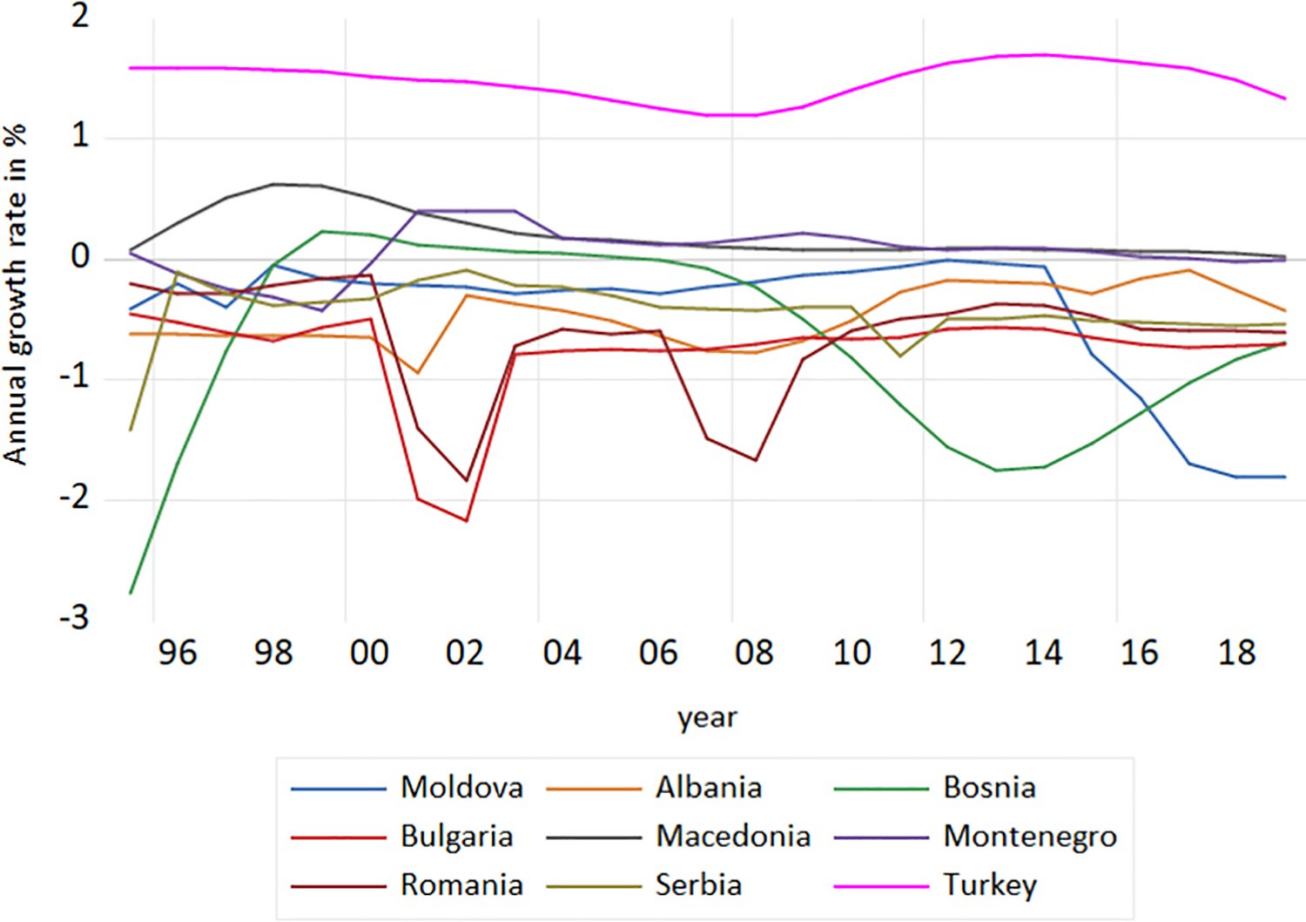
Population growth rate in the European middle income countries, 1995–2019. **Source:** Author’s design based on World Bank and UN data.

The trend in population growth is very likely to be associated with the trend in ageing process. As claimed by [31], the phase of old age is part of the life course, and hence the ageing process is a part from demographic developmental changes. The level of population growth may be linked with migration trends as well. Some European countries, mostly in Eastern and Southern Europe are faced with emigration, in many cases of their working young population with little immigration or without any influx of migrants from other countries [12]. Thus, comparing different societies and cultures seems especially useful for the analysis of societal and cultural factors in life course development. The *population growth rate* in the countries under the study is shown in Fig 4. Ageing is determined not only by the pace of growth of the older population, but also by the growth rates of the other age groups, i.e., the growth of the entire population [23]. In fact, as specified by [5], the improvements in life expectancy at the early stage of life rejuvenated the population, because the early age increases in life expectancy have been resulted by a decline in infant and child mortality. Consequently, most of the actual years gained through these increases were below the age of 65, but for the Western countries these gains referred above 72 years.

## Data and methods

Data on age-specific mortality rates, population growth and population ages above 65+ for nine low and middle income European countries during the 25 years of time period (1995–2019) were obtained from the World Bank database (https://data.worldbank.org/indicator) and UN databases (https://population.un.org/wpp/DataQuery/) [32–35]. Because of the absence of data for the adult mortality rate for Bulgaria for 2018 within the World Bank database, the data for Bulgaria regarding adult mortality rate in 2018 were obtained from the UN mortality estimations. Furthermore, in order for our sample to be up to date with the newest data, due to the absence of data on adult mortality rate for both sexes and by sex for 2019 within World Bank database, therefore, the data on adult mortality for 2019 are obtained from the latest UN publications on mortality statistics. In order to examine the data at comparable level, the focus is on the regression model for the pooled time series with cross-section fixed effects. The pool feature allows analyzing multiple series observed for the same variable for a number of countries. The data have been pooled in a regression with common coefficients for all countries as well as for individual coefficients by building cross-section fixed effect estimators. The interpretation of R-squared and *F* statistics is that they describe the explanatory power of the entire specification, including the estimated fixed effects. Besides the number of parameters and the number of estimated coefficients, the reported information criterion includes fixed effects. The reported Durbin-Watson statistics is formed simply by computing the first-order residual correlation on the stacked set of residuals. In addition, the Fixed/Random Effects Testing/Redundant Fixed Effects-Likelihood Ratio was used to test for country specific intercepts against a single, common, intercept. Finally, a Pool unit root test was estimated to test if our model is stationary.

The novelty within the empirical part and in the research work as well is that our research uses the White two-way cluster method for clustering by both cross-section and period. In many examples, it is known that observations may be grouped in different groups or “clusters” where errors are correlated for observations in the same cluster and uncorrelated for observations in different clusters. Therefore, while computing the estimates of accuracy of regression estimates this cluster error correlation must be considered. Having in mind the influential system estimation literature, these robust standard error calculations have been designated “White cross-section” for clustering by period, to indicate that there were contemporaneous correlation between cross-section units, and also labeled “White period” for clustering by cross-section, to indicate that there was between period correlations within a cross-section unit [36]. The research study extends these tools in order to allow for computation of robust covariance when clusters are defined by both cross-section units and periods, i.e. the White two-way cluster method was used [36].

Furthermore, the Ordinary least squares method of estimating the parameters of the system of equations was used. Each equation in the system was estimated separately, using the Ordinary least squares. Ordinary least squares minimize the sum-of-squared residuals for each equation, considering any cross-equation restrictions on the parameters of the system. If there are no any restrictions, this method is exactly (the same) to estimating each equation using single-equation ordinary least squares [37]. The OLS estimator of the estimated variance matrix of the parameters is credible under the presumption that *V* = *Σ*⊗*I*_*T*_. The estimator for *β* has been given by Eq (1):

$$b_{LS}={\left(X^{\prime }X\right)}^{-1}X^{\prime }y$$ (1)

and the variance estimator has been specified in Eq (2):

$$var(b_{LS})=s^{2}{\left(X^{\prime }X\right)}^{-1}$$ (2)

where *s*^2^ is the residual variance estimate for the system [37].

## Application of pooled least squares model and main findings

The model was estimated using a pool object with cross-section coefficients. Since all of the *β*_*it*_ coefficients are common across cross-sections and periods, there are a total of *k* coefficients in *β*, each corresponding to an element of *x* [38]. The specification may be written as:

$$Y_{it}=\alpha +X_{it}^{\prime }\beta +\delta_{i}+\gamma_{t}+\epsilon_{it},$$ (3)

Where *Y*_*it*_ is a dependent variable and *X*_*it*_ is a *k* -vector of regressors, and *ϵ*_*it*_ are the error terms for *i* = 1, 2,…, *M* cross-sectional units observed for dated periods *t* = 1, 2,.., *T*. The *α* parameter represents the overall constant in the model, while the *δ*_*i*_ and *γ*_*t*_ represent cross-section or period specific effects (random or fixed). A balanced country-level stacked data that contains annual observations on proportion of population above 65+ (POP65+), adult mortality rate for both sexes (MRAB), population growth rate (POPG) and under5 mortality rate (UNDER5) have been used within our model. A model regressing POP65 on the common regressors MRAB, POPG and UNDER5 was estimated with a cross-section fixed effects. In this regression the data for nine low and middle income European countries were combined. The estimation is given by the following Table 1:

**Table 1:** Estimation results for the pooled series with cross-section fixed effects.

Variable	Coefficient	Std. Error	t-Statistic	Prob.
C	21.971	1.9873	11.056	0.0000
MRAB	0.1099	0.0364	3.0179	0.0166
POPG	-0.1018	0.0224	-5.5380	0.0019
UNDER5	-0.0609	0.0818	-0.7449	0.4476
Fixed effects (Crossed)				
MOLDOVA_C	4.0685			
ALBANIA_C	-6.2010			
BOSNIA_C	-1.5645			
BULGARIA_C	8.1251			
MACEDONIA_C	-3.8415			
MONTENEGRO_C	-2.1461			
ROMANIA_C	6.1017			
SERBIA_C	1.4400			
TURKEY_C	-5.9821			
Effects specifications				
Root MSE	0.9281	R-squared		0.9334
Mean dependent var	12.424	Adjusted R-squared		0.9300
S.D. dependent var	3.6060	S.E. of regression		0.9539
Akaike info criterion	2.7954	Sum squared resid		193.82
Schhwarc criterion	2.9776	Log likelihood		-302.48
Hannah-Quinn crit.	2.8689	F-statistic		271.63
Durbin-Watson stat	0.1297	Prob(F-statistic)		0.0000

**Dependent variable**: POP65+

**Method**: Pooled Least Squares

**Sample**: 1995–2019. Included observations: 25

**Cross-sections** included: 9. **Total** (pool) balanced **observations**: 225.

**White two-way cluster standard errors & covariance (d.f.corrected)

** Standard error and t-statistic probabilities adjusted for clustering

**Source:** Author’s calculations.

**Fig 5:**
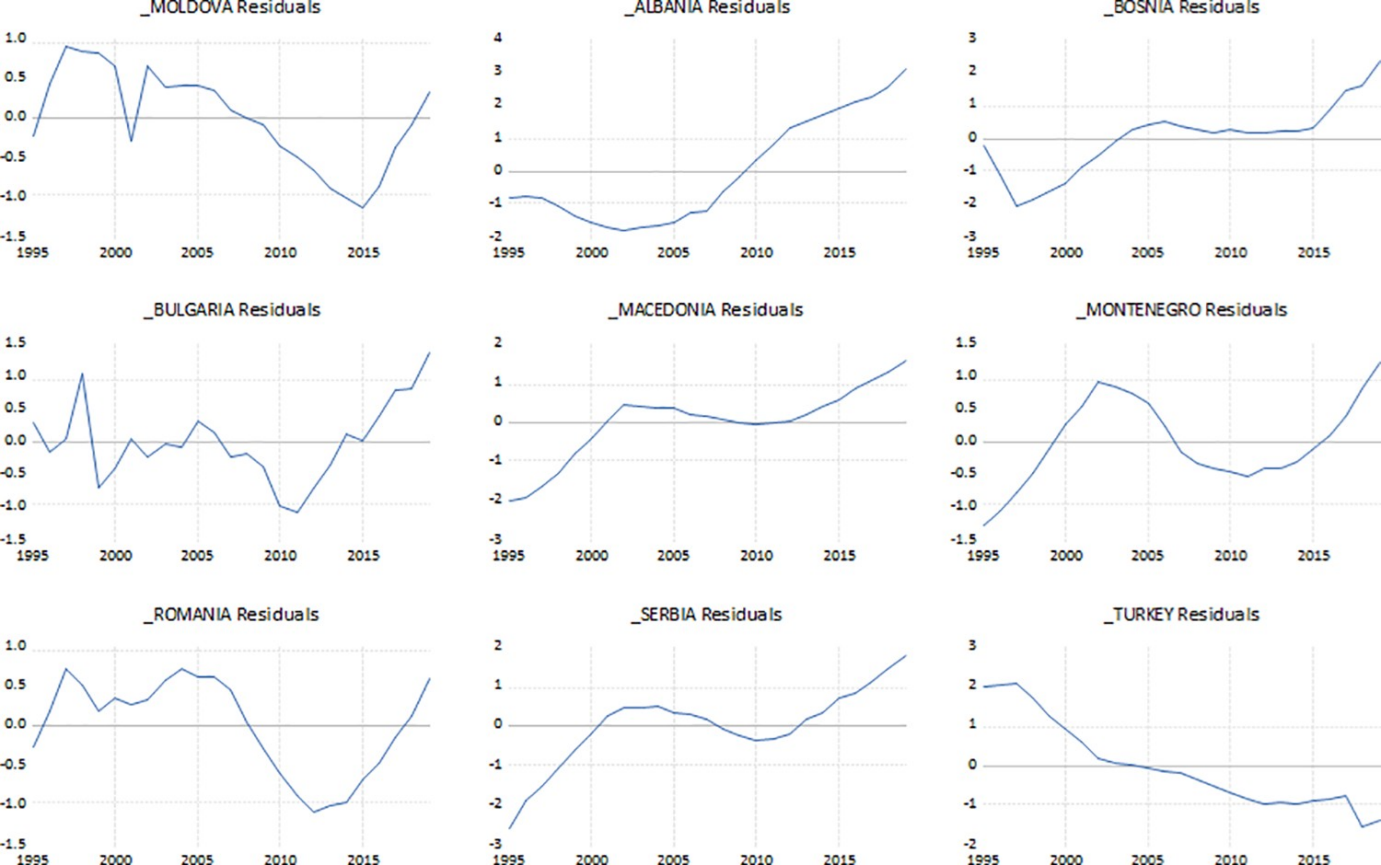
Fixed effect residual country plot graph. **Source:** Author’s design based on World Bank and UN data.

The summary of pool unit root tests, as the LLC (Levin, Lin & Chu t*), and both Fisher tests (ADF—Fisher Chi-square and PP-Fisher Chi-square) indicate to the presence of a common and individual unit root process, i.e. fail to reject the null of a unit root. The cross-section specific constant picks up all the things that make one country different from another that are not included in the model. Country-specific constants are called *fixed effects* [39]. The intercept is reported in two parts, the first “C” in Table 1 reports the average value of the intercept for all the countries in the sample. The lines marked for the individual countries give the country’s intercept as a deviation from that overall average. In Table 1 the overall average intercept has a positive value, i.e. 21.97 and the intercept for the different countries is positive and negative. From Table 1 it can be seen clearly that the fixed effects for Moldova, Bulgaria, Romania and Serbia are positive and the effects for Albania, Bosnia and Herzegovina, Macedonia, Montenegro and Turkey are negative. These results mean that the intercept for Bulgaria, for example, is 30.1 (8.12 above the average intercept, 21.97). On the other side, the intercept for Albania is 15.77 (6.20 below the average intercept, 21.97). Choosing the Redundant Fixed Effects-Likelihood Ratio test as shown by its *p*-value in the output, it can be seen that the statistical evidence is overwhelmingly in favor of keeping fixed effects in our model. The values of Cross-section F (132.45) and Cross-section Chi Square (402.20) as well as its associated *p*-values (0.0000) strongly reject the null that the cross-section effects are redundant. With the cross-section F and Chi square tests the hypothesis of a common intercept is rejected and thus strongly confirmed the fixed effect specification. By specifying fixed effects each country’s residual plot can be seen from Fig 5. From Fig 5 it is observable that almost each of the country’s residuals is not centered on zero. The biggest deviations are observed for Albania, Macedonia, Serbia and Turkey.

From the results of the pool regression in Table 1 it can be noticed that all coefficients except the one of the under-five mortality rate are statistically significant at 5% level of significance. The adult mortality rate for both sexes has a positive effect and population growth rate has a negative effect on the proportion of population 65+. However, the *F* test confirms the joint significance of variables that are included in the pool. The results estimate that the MRAB, POPG and UNDER5 are statistically significant variables within the model. Thus, lower adult mortality rate in both sexes lead to lower proportion of population 65+ suggesting that the people in all nine countries are relatively entering the ageing process. The population growth variable has a negative sign and the effect of the lower population growth appears to have increased the proportion of population 65+. The same explanation could be given for the effect of the mortality under-five on proportion of population 65+, but this effect is insignificant.

In addition, these findings can be explained and discussed further with the causality results that were obtained from. Thus, the direction of causality between population ageing and age specific mortality rates has attracted a lot of interest in empirical studies. This research study investigates the direction of causality issues employing Pairwise Granger Causality Tests with two lags. The number of lags has been selected using Akaike Information criterion as well as final Prediction Error criterion. The evidence indicates only the two-way directional causality that goes from adult mortality rate for both sexes to the population 65+ and *vice versa*. The first directional causality, i.e. the causality from adult mortality rate for both sexes to the population 65+ was found at 5% level, and the second directional causality from the population 65+ to the adult mortality rate for both sexes was revealed at 10% level. A one-way directional causality from population 65+ to population growth and also from population 65+ to under-five mortality was found within our pooled model but at 10% level.

So far, the estimation has been based on one equation. Furthermore, system estimation has been applied, which estimates jointly the parameters of more equations. Since the research purpose was to assess the estimated coefficients that have different values in more than one equation (e.g. for different countries), system estimation was the only worthy way to go with it. The same coefficient name for each country was used in each equation that was specified. Choosing Ordinary Least Squares the estimates for nine equations, i.e. nine countries have been generated. The estimation was made for equation-by-equation ordinary least squares. The results of system estimation are provided in Tables 2–5.

**Table 2:** System estimation coefficients: Ordinary least squares.

System estimation method: Least squares				
Country’s variables	Coefficient	Std. Error	t-Statistic	Prob.
Moldova_Intercept (C1)	19.20	3.38	5.69	0.00
Moldova_MRAB (C2)	0.07	0.04	1.93	0.06
Moldova_POPG (C3)	-0.06	0.03	-2.15	0.03
Moldova_UNDER5 (C4)	-0.05	0.02	-3.02	0.00
Albania_Intercept (C5)	19.44	4.27	4.55	0.00
Albania_MRAB (C6)	-0.29	0.14	-2.10	0.04
Albania_POPG (C7)	0.10	0.04	2.28	0.02
Albania_UNDER5 (C8)	-0.20	0.09	-2.22	0.03
Bosnia_Intercept (C9)	36.37	1.42	25.70	0.00
Bosnia_MRAB (C10)	-0.39	0.05	-7.66	0.00
Bosnia_POPG (C11)	-0.02	0.03	-0.62	0.53
Bosnia_UNDER5 (C12)	0.97	0.19	5.10	0.00
Bulgaria_Intercept (C13)	26.97	3.92	6.88	0.00
Bulgaria_MRAB (C14)	0.02	0.06	0.38	0.70
Bulgaria_ POPG (C15)	-0.04	0.02	-1.71	0.09
Bulgaria_UNDER5 (C16)	-0.24	0.10	-2.45	0.02
Macedonia_Intercept (C17)	22.63	0.61	37.37	0.00
Macedonia_MRAB (C18)	-0.07	0.02	-3.10	0.00
Macedonia_ POPG (C19)	-0.03	0.01	-3.18	0.00
Macedonia_UNDER5 (C20)	-0.06	0.03	-2.00	0.05
Montenegro_Intercept (C21)	-1.75	4.58	-0.38	0.70
Montenegro_ MRAB (C22)	-0.05	0.05	-0.87	0.38
Montenegro_POPG (C23)	0.17	0.06	2.69	0.01
Montenegro_UNDER5 (C24)	-0.90	0.17	-5.28	0.00
Romania_Intercept (C25)	24.28	3.69	6.57	0.00
Romania_MRAB (C26)	-0.13	0.13	-0.99	0.32
Romania_POPG (C27)	0.02	0.05	0.40	0.69
Romania_UNDER5 (C28)	-0.06	0.16	-0.38	0.70
Serbia_Intercept (C29)	17.98	1.63	11.03	0.00
Serbia_MRAB (C30)	0.56	0.10	5.68	0.00
Serbia_POPG (C31)	-0.27	0.04	-6.86	0.00
Serbia_UNDER5 (C32)	-0.63	0.08	-8.16	0.00
Turkey_Intercept (C33)	14.41	0.64	22.59	0.00
Turkey_MRAB (C34)	-0.07	0.03	-2.41	0.02
Turkey_POPG (C35)	-0.02	0.01	-1.20	0.23
Turkey_UNDER5 (C36)	0.04	0.01	3.07	0.00

**Source:** Author’s calculations.

**Table 3:** System estimation by country equation.

Equation: POP65_Moldova = C(1)+C(2)*MRAB_ Moldova +C(3)*POPG_ Moldova+ C(4)*UNDER5_Moldova			
Observations: 25			
R-squared	0.70	Mean dependent var	10.02
Adjusted R-squared	0.65	S.D. dependent var	0.68
S.E. of regression	0.40	Sum squared resid	3.39
Durbin-Watson stat	0.57		
Equation: POP65_Albania = C(5)+C(6)*MRAB_ Albania +C(7)*POPG_ Albania+ C(8)*UNDER5_Albania			
Observations: 25			
R-squared	0.93	Mean dependent var	9.68
Adjusted R-squared	0.92	S.D. dependent var	2.54
S.E. of regression	0.74	Sum squared resid	11.53
Durbin-Watson stat	0.16		
Equation: POP65_Bosnia = C(9)+C(10)*MRAB_ Bosnia +C(11)*POPG_ Bosnia+ C(12)*UNDER5_Bosnia			
Observations: 25			
R-squared	0.98	Mean dependent var	13.14
Adjusted R-squared	0.98	S.D. dependent var	2.13
S.E. of regression	0.29	Sum squared resid	1.79
Durbin-Watson stat	1.10		

*Notes*:**C(1) = Intercept Moldova; C(5) = Intercept Albania; C(9) = Intercept Bosnia

**Source:** Author calculations.

**Table 4:** System estimation by country equation.

Equation: POP65_Bulgaria = C(13)+C(14)*MRAB_ Bulgaria +C(15)*POPG_ Bulgaria+ C(16)*UNDER5_Bulgaria			
Observations: 25			
R-squared	0.92	Mean dependent var	18.00
Adjusted R-squared	0.91	S.D. dependent var	1.75
S.E. of regression	0.53	Sum squared resid	5.85
Durbin-Watson stat	0.26		
Equation: POP65_Macedonia = C(17)+C(18)*MRAB_ Macedonia +C(19)*POPG_ Macedonia+ C(20)*UNDER5_Macedonia			
Observations: 25			
R-squared	0.98	Mean dependent var	11.21
Adjusted R-squared	0.98	S.D. dependent var	1.47
S.E. of regression	0.23	Sum squared resid	1.08
Durbin-Watson stat	0.30		
Equation: POP65_Montenegro = C(21)+C(22)*MRAB_ Montenegro +C(23)*POPG_ Montenegro+ C(24)*UNDER5_Montenegro			
Observations: 25			
R-squared	0.91	Mean dependent var	12.71
Adjusted R-squared	0.90	S.D. dependent var	1.43
S.E. of regression	0.46	Sum squared resid	4.43
Durbin-Watson stat	0.28		

*Notes*: **C(13) = Intercept Bulgaria; C(17) = Intercept Macedonia; C(21) = Intercept Montenegro.

**Source:** Author calculations.

**Table 5:** System estimation by country equation.

Equation: POP65_Romania = C(25)+C(26)*MRAB_ Romania +C(27)*POPG_ Romania+ C(28)*UNDER5_Romania			
Observations: 25			
R-squared	0.94	Mean dependent var	15.30
Adjusted R-squared	0.93	S.D. dependent var	1.82
S.E. of regression	0.47	Sum squared resid	4.67
Durbin-Watson stat	0.24		
Equation: POP65_Serbia = C(29)+C(30)*MRAB_ Serbia +C(31)*POPG_ Serbia+ C(32)*UNDER5_Serbia			
Observations: 25			
R-squared	0.97	Mean dependent var	14.83
Adjusted R-squared	0.96	S.D. dependent var	1.95
S.E. of regression	0.38	Sum squared resid	3.08
Durbin-Watson stat	0.52		
Equation: POP65_Turkey = C(33)+C(34)*MRAB_ Turkey +C(35)*POPG_ Turkey+ C(36)*UNDER5_Turkey			
Observations: 25			
R-squared	0.99	Mean dependent var	6.92
Adjusted R-squared	0.98	S.D. dependent var	0.97
S.E. of regression	0.12	Sum squared resid	0.30
Durbin-Watson stat	1.11		

*Notes*: **C(25) = Intercept Romania; C(29) = Intercept Serbia; C(33) = Intercept Turkey

**Source:** Author calculations.

## Discussion of the results

The results from Table 1 show that the coefficient of the adult mortality rate is an estimate of the long-run adult mortality elasticity, and with positive and significant effect on proportion of population 65+ at 5% level. It is as suggested by the second demographic transition theory and to some other approaches of the scholar’s expectations as well. The long run population growth elasticity is significant at 5% and even to 1% level. In other words, the estimated *p*-value of this coefficient showed that it is statistically significant and with a negative effect on the proportion of population 65+. The empirical results show that population growth can contribute to accelerated ageing of the population. This shows that as the study countries continue to have zero growth or negative population growth, their proportion of population over 65 will increase more rapidly in the future. The value of the under-five mortality coefficient is statistically insignificant at 5% level and negative. This finding is not consistent with the second demographic transition theory.

Table 1 shows that all estimated coefficients are not so significantly different from zero, except, the estimated coefficient of the overall intercept. This may imply a stable ageing acceleration within these countries. This is also confirmed by [20] discussion who indicated that, “the major declines in mortality have had little effect on the basic rate of mortality acceleration during ageing”. In other words, despite the trend in overall mortality across cohorts, the mortality slope (Gompertz slope) appeared to be relatively stable.

Moreover, the research results obtained are in line with the mechanism between mortality, population growth and ageing process of the population explained by [4]. Accordingly, with this operating mechanism, it was given a clear response to the one of the major questions that was set up for this research: Does mortality acceleration in older ages is weak when young-age mortality is high and strong when young-age mortality is low? In addition, this mechanism also indicated that the rate of demographic ageing in fact is negatively affected by young-age mortality. And, finally, following this mechanism, according to the obtained empirical results there should be a clear answer to the last research question that was set up: What demographic events have driven the changes in the rate of demographic ageing? Thus, these events are: decreasing of both population growth and adult mortality level and the past and current levels of under-five mortality rate.

From the results of pooled model estimation it is observable that demographic developments in terms of mortality changes of both under-five mortality and by the trend and variation changes of adult mortality for both sexes have supported population ageing in these countries. In addition, the mechanism between mortality, population growth and ageing process of the population is explained especially by the levels of population growth. It would be expected that the pace of population ageing will continue in the coming decades, most likely with slow speed of ageing as was shown by the measures that have been used and it would probably be completed before the end of this century. But, however, it does not mean that all of the problems related to population ageing will disappear. Hence, the adjusting to population ageing process probably will remain to be a challenging and demanding issue within these European middle income countries that are subject of our research work. Accordingly, the major hypotheses that were set up for this research that: “There is association between mortality, population growth and population ageing in the European LUMIs” and that: ‘‘Mortality acceleration in older ages within these countries is affected by the main demographic events”, are therefore clearly confirmed.

From the individual results for each country’s coefficients separately within the system estimation (Table 2), it can be seen a significant positive effect of the UNDER5 and MRAB coefficients on the proportion of the population 65+ in Bosnia and Herzegovina (+0.97) and Serbia (+0.56), respectively. Most of the effects on the proportion of the population 65+ or the aging process in the population are negative with bigger or lesser effects. The biggest negative effect on the proportion of population 65+ is from the impact of UNDER5 in Montenegro (-0.90) and Serbia (-0.63) and the impact of MRAB in Bosnia and Herzegovina (-0.39). It is characteristic that the impact of the intercept coefficient on the proportion of the population 65+ in all countries is very high and positive (Table 2), except for Montenegro, where the impact of this coefficient on the proportion of the population 65+ is negative and is the lowest (-1.75). If looked at Tables 3–5 it can be noticed that the highest R-squared have Turkey, Macedonia, Bosnia and Herzegovina and Serbia (over 0.97). The lowest coefficient of R-squared is observed for Moldova (0.70). In this respect, Montenegro, Bulgaria, Albania and Romania are somewhere in the middle.

## Concluding remarks

The current study contributes to the ageing-mortality-population growth link focusing on nine purposively selected European low and middle income European countries using pooled model. In addition, the empirical findings are valuable in terms showing that population growth has demonstrated a negative relationship with proportion of population 65+, i.e. as an indicator for the population ageing. The challenges associated with population ageing within these countries are now beginning to be the most important. Given the robustness of the model explained in Section 4, it may be considered as relevant and could serve as a solid basis for policy makers in the respective countries. Because obviously the population in these countries will continue to grow older, then in this case, policy-makers should consider the options on how to adjust to this continuous ageing process. It is very important for policy programs to be adjusted with respect to socioeconomic development. It is expected that the effects of population ageing differ by country, as they depend on each country’s specific demographic history and implementation of policy objectives. Anyhow, from the perspective of the second demographic transition theory it will be more advisable for these countries to implement social policies and programs that make social security more accessible and affordable to older people. Furthermore, what is more important is that these countries benefit from implementing social policy reforms and more favorable economic circumstances for the youth and households, in order to prevent future social issues. This is really very important, given ageing process within these mentioned nine European low and middle income countries.

## Supporting information

S1 Data (XLS)
